# Multinucleated giant cells within the in vivo implantation bed of a collagen-based biomaterial determine its degradation pattern

**DOI:** 10.1007/s00784-020-03373-7

**Published:** 2020-06-08

**Authors:** Anna Maria Tanneberger, Sarah Al-Maawi, Carlos Herrera-Vizcaíno, Anna Orlowska, Alica Kubesch, Robert Sader, C. J. Kirkpatrick, Shahram Ghanaati

**Affiliations:** Department for Oral, Cranio-Maxillofacial and Facial Plastic Surgery, FORM (Frankfurt Orofacial Regenerative Medicine) Lab, University Hospital Frankfurt Goethe University, Theodor-Stern-Kai 7, 60590 Frankfurt am Main, Germany

**Keywords:** Guided tissue regeneration (GTR), Guided bone regeneration (GBR), Inflammatory pattern, Integration, Disintegration

## Abstract

**Objectives:**

The aim of the present study was to characterize the cellular reaction to a xenogeneic resorbable collagen membrane of porcine origin using a subcutaneous implantation model in Wistar rats over 30 days.

**Materials and methods:**

Ex vivo, liquid platelet-rich fibrin (PRF), a leukocyte and platelet-rich cell suspension, was used to evaluate the blood cell membrane interaction. The material was implanted subcutaneously in rats. Sham-operated rats without biomaterial displayed physiological wound healing (control group). Histological, immunohistological, and histomorphometric analyses were focused on the inflammatory pattern, vascularization rate, and degradation pattern.

**Results:**

The membrane induced a large number of mononuclear cells over the observation period, including lymphocytes, macrophages, and fibroblasts. After 15 days, multinucleated giant cells (MNGCs) were observed on the biomaterial surface. Their number increased significantly, and they proceeded to the center of the biomaterial on day 30. These cells highly expressed CD-68, calcitonin receptor, and MMP-9, but not TRAP or integrin-ß3. Thus, the membrane lost its integrity and underwent disintegration as a consequence of the induction of MNGCs. The significant increase in MNGC number correlated with a high rate of vascularization, which was significantly higher than the control group. Physiological wound healing in the control group did not induce any MNGCs at any time point. Ex vivo blood cells from liquid-PRF did not penetrate the membrane.

**Conclusion:**

The present study suggests a potential role for MNGCs in biomaterial degradation and questions whether it is beneficial to accept them in clinically approved biomaterials or focus on biomaterials that induce only mononuclear cells. Thus, further studies are necessary to identify the function of biomaterial-induced MNGCs.

**Clinical relevance:**

Understanding the cellular reaction to biomaterials is essential to assess their suitability for specific clinical indications and outline the potential benefit of specific group of biomaterials in the respective clinical indications.

## Introduction

The principle of guided tissue regeneration (GTR) and guided bone regeneration (GBR) is based on the separation of different cells and tissues that compete during the healing process [1, 2]. Different collagen-based biomaterials have been utilized in oral and maxillofacial surgery to promote healthy soft tissue regeneration (GTR) or bone regeneration (GBR) while inhibiting the ingrowth of undesirable fibrotic tissue into the bony defect [1, 3–5]. In addition to biocompatibility and suitability in clinical handling, membranes are required to fulfill a so-called barrier function and to act as a place holder [1, 6, 7].

Xenogeneic collagen-based membranes were used successfully in GTR/GBR methods [8–10]. Collagen is an ubiquitous protein in human and animal tissue that undergoes enzymatic degradation via matrix metalloproteases (MMP) released by neutrophils, monocytes/macrophages, eosinophils, and fibroblasts recruited during wound healing [11, 12]. In addition to the angiogenic potential of collagen type I, it plays an important role in tissue regeneration [7, 13, 14].

Currently a wide range of different collagen-based biomaterials is available for clinical applications [15]. However, the regenerative mechanisms of the membranes are still not fully explored [16]. Additionally, different manufacturing and processing techniques influence the biomaterials’ specific surface and physicochemical properties such as porosity, polarity, and hydrophilicity. Accordingly, the biomaterial attracts a different type of cellular reaction [17, 18]. Previous studies have shown different collagen-based materials of the same origin, i.e., porcine induce different cellular reactions according to their properties after their implantation in vivo [19, 20]. In this context, the formation of biomaterial-induced multinucleated giant cells (MNGCs) has been frequently observed [21–24]. Different studies discussed the morphological and possible functional similarities of these cells to physiologically existing osteoclasts and disease-related MNGCs [21, 25, 26]. Further studies showed that their formation leads rather to a premature biomaterial disintegration and enhanced vascularization in vivo [19, 23]. However, the reason for their formation and the role of these cells in the biomaterial-based regeneration process are still unexplored.

This study aimed to analyze the cellular reaction of a resorbable membrane consisting of collagen and elastin. Their combination in an interwoven structure is thought to provide the membrane a high in vivo stability. This novel collagen-based membrane, Creos™ Xenoprotect (CXP, Nobel Biocare, Gothenburg, Sweden; Remaix, Matricel GmbH, Herzogenrath, Germany), is derived from porcine collagen and elastin, which are manufactured to a highly purified non-cross-linked collagen membrane. Special attention was paid to the cellular response and degradation pattern of the specifically reinforced collagen membrane CXP. Additionally, this study evaluated the biomaterial-induced MNGCs and their differentiation. Moreover, liquid platelet-rich fibrin (PRF), a blood concentrate system obtained from the peripheral blood, was used to examine the initial interaction between collagen and fibrin, including the physiological existence of blood cells derived from peripheral blood to analyze the initial permeability of the biomaterial ex vivo.

## Materials and methods

### Creos™ Xenoprotect (CXP)

According to the manufacturer, CXP is a xenogeneic, biodegradable, non-chemically cross-linked collagen membrane of porcine origin, which is indicated in clinical cases requiring GBR and GTR. This collagen membrane is marked as *Conformité Européene* (CE). The hydrophilic membrane is composed of collagen and elastin fiber networks, which supply it with high mechanical strength and avoid invasion of the surrounding tissue.

### Ex vivo analysis of the biomaterial-blood interaction

PRF is a blood concentrate system that is produced by the centrifugation of human peripheral blood. PRF is composed of platelets, leukocytes, and plasma proteins. In this study, PRF was used as a cell suspension to evaluate the membrane-cell interaction and occlusion. The ex vivo analysis was performed as previously described [22, 23]. Three healthy volunteers participated in this study, and informed consent was obtained before blood withdrawal according to a local ethical approval (265/17). Using a 24-gauge butterfly needle, 10 ml of blood was withdrawn from the antecubital vein direct into 10-ml liquid-PRF tubes covered with plastic in the interior (Process for PRF, Nice, France). The tube was quickly transported to a tabletop centrifuge (Duo centrifuge, Process for PRF, Nice, France (11 cm rotor, fixed angel)) and processed at low speed to allow the concentration of blood cells in high numbers using a relatively low centrifugation force (44×*g* and 8 min) [27]. The blood was fractioned into a superior liquid segment containing liquid-PRF and a lower segment of erythrocytes. The superior segment was collected using a 1-ml pipette tip and transported in 5-ml plastic tubes for homogenization. Concomitantly, the Creos™ Xenoprotect (CXP) membranes were cut into 1-cm^2^ segments and placed in 24-well cell culture plates. Before coagulation of liquid-PRF, 1 ml was deposited on top of the membranes until they were covered and kept at room temperature for 15 min until clot formation. The samples were fixed with formaldehyde (*Roti-Histofix* 4% acid free pH 7, Carl-Roth, Germany) over 24 h for histological evaluation.

### In vivo analysis of the cellular reaction

The design, analysis, and reporting of the experiments were conducted following the ARRIVE guidelines for animal research [28]. The experiments were approved by the government regulating agency of Darmstadt and the ethical committee from the University of Goethe of Frankfurt am Main (FK/1023). Animal husbandry, care, and surgeries were carried out in the animal care facility ((ZFE) Frankfurt am Main, Germany) of the Department of Medicine of Johann Wolfgang Goethe University Frankfurt. Twenty-four female Wistar rats (*Rattus norvegicus*) were purchased from Charles River (Sulzfeld, Germany) with an average age of 7–8 weeks and weighing between 190 and 220 g. The experiments were performed in summer 2016. The animals were housed in groups for 1 week prior to surgery for acclimatization in a controlled environment (temp, 20 °C; light/dark cycles of 12 h; and humidity of 40% to 70%) and fed regular rodent pellets and water ad libitum. The animals were provided from Charles River in groups of 4 in one cage and were prepared by an independent person from the animal facility. In sequence, animals in each cage (*n* = 4) were alternately used either for implantation (test group) or for the control group. The surgical intervention was performed following a standardized and established model as previously described [29, 30]. Anesthesia was induced through intraperitoneal injection of a mixture of ketamine/xylazine (100 mg/kg/5 mg/kg). The animals were placed in the prone position, and the depth of anesthesia was verified by the absence of the toe reflex. A subcutaneous pocket was prepared by generating a 2-cm incision in the rostral skin and dissection of the subcutaneous tissue into the interscapular area. The biomaterial was cut in segments of 1 cm^2^ and placed above the muscle fascia. The tissue was sutured using a 4–0 polypropylene suture (Prolene Ethicon). Tramadol (1–3 mg/kg/d) was administered orally for pain management through the drinking water for 1 day. Four animals were included in the experimental group, and four served as controls (sham surgery) to evaluate the physiological wound healing per time point. In the control group of animals, anesthesia and surgery were performed as described for the experimental group, but without the implantation of a biomaterial. Tissue explantation involving the biomaterial and the surrounding implantation bed was performed on day 3, 15, and 30 and fixed with formaldehyde (*Roti-Histofix* 4% acid free pH 7, Carl-Roth, Germany) over 24 h for histological evaluation.

### Tissue processing and histological preparation

Histological preparation was performed as previously described [31, 32]. The fixed tissue mentioned above was segmented and distributed in embedding cassettes (Histosette, VWR, Deutschland) before processing in increasing concentrations of alcohol and xylol, followed by the infiltration of paraffin wax under a vacuum using an automatic tissue processor (Leica TP1020). After processing, the samples were embedded in paraffin blocks, and slides from all blocks with a thickness of 3 μm were obtained from consecutive sections using a rotation microtome (Leica RM2255) and stained with hematoxylin and eosin for screening. The block containing the most representative sagittal section of the biomaterial was selected, and nine consecutive slides were obtained and stained as described previously; the first four slides were stained with Mayer’s hematoxylin and eosin (H&E) (1), Azan (2), Masson-Goldner (3), and TRAP (4). The remaining five slides were immunohistochemically stained using an autostainer (Lab Vision™ Autostainer 360, Thermo Scientific, Germany). Antigen retrieval was performed using the heat-induced epitope retrieval (HIER) method. The slides were immersed in citrate buffer, pH 6.0, and heated using a water bath (VWR®, Germany) at 95 °C for 20 min. Subsequently, after protein blocking (Avidin/Biotin Blocking Kit, Vector Laboratories, USA), antigens were immunodetected with mouse anti-rat CD-68 monoclonal antibody (Bio-Rad; MCA341GA; concentration 1:400; 30 min) as a pan marker for the monocyte lineage (5). Goat anti-rabbit IgG-B (Santa Cruz Biotechnology, USA. sc-2040; 1:200; 60 min) was used as the secondary antibody with further amplification of the antigen using the avidin-biotin complex method (ABC, Thermo Fisher Scientific, Germany). Finally, the slides were stained with α-smooth muscle actin (α-SMA) (Sigma-Aldrich, Germany; A5228; concentration 1:20.000; 120 min) for vascular endothelial cells (6), MMP-9 (ab58803, 1: 250) (7), calcitonin receptor (ab11042, 1:200) (8), integrin-β3 (ab225742, 1:2000), and (9) followed by anti-mouse secondary antibody (HRP Ultravision Quanto Detection System, Thermo Fisher Scientific). Chromogenic visualization was obtained by applying AEC (3-amino-9-ethylcarbazole) peroxidase (Thermo Fisher Scientific, Germany) for CD-68 and α-SMA or DAB (diaminobenzidine (DAB) Quanto chromogen and substrate) for calcitonin receptor, integrin-β3 and MMP-9 and counterstaining with Mayer’s hematoxylin. From the set of samples, two supplementary, randomly selected slides were added as a negative control without application of the primary antibody. For immunohistochemical staining, rat bone was used as positive control. As negative controls, the primary antibodies were replaced by antibody substrate and used to stain rat bone as described.

### Qualitative histological evaluation

The stained slides were systematically evaluated by the authors (AT, SA, and SG) using a light microscope (Nikon Eclipse 80i, Tokyo, Japan). The cellular reaction in the implantation bed, characteristics of the biomaterial, and vascularization were compared within the experimental group (intracomparison) at all time points and with the control group (intercomparison). Representative histological images were captured with a Nikon DS-Fi1 digital camera and a Nikon Digital sight unit DS-U3 (Nikon, Tokyo, Japan), which were controlled using Nikon’s NIS-Elements imaging software (Nikon, Tokyo, Japan). Six samples per animal were stained from which two samples per animal from the center region were used for histomorphometry.

### Quantitative histological analysis

Histomorphometry was performed following established parameters as previously described [29, 33]. All stained slides were scanned with the Nikon ECLIPSE 80i histological microscope at high magnification using the large image settings in the NIS-Elements software (Nikon, Tokyo, Japan). Using an automatically motorized stage, a total of 100–200 images were obtained at × 200 for each sample and used to construct the “total scan,” allowing complete visualization of the sample and histomorphometric analyses.

#### Measurement of vascularization

After obtaining the “total scan” of α-SMA positively stained structures on day 3, 15, and 30, the vascular structures in the implantation bed and within the biomaterial were manually counted using the annotation and measurement function in the NIS-Elements software (Nikon, Tokyo, Japan). To obtain different perspectives regarding the angiogenesis process during implantation of the biomaterial, the quantified vascular structures were first expressed as the density and then calculated by dividing the total vascular structures by the total area of the sample (vessel/mm^2^). Second, the lumen area of the vascular structures was quantified and expressed as a percentage (%).

#### Quantification of multinucleated giant cells (MNGCs) and CD-68-positive mononuclear cells

As described previously, the slides were scanned using the large image capture tool (“total scan”) in the Nis element software (Nikon, Tokyo, Japan). The positively stained MNGCs and their subtypes, TRAP positive or TRAP negative, as well as CD-68-positive monocytes were quantified using the manual “annotations and measurements” tool in the NIS-Elements software. For each individual slide, the total number of counted cells was divided by the total area of the implantation bed as a relative measurement to evaluate the cellular reaction of the Creos™ Xenoprotect (CXP) membrane. The results are expressed as MNGCs/mm^2^ (TRAP-/TRAP+) and CD-68-positive monocytes/mm^2^, and they were statistically evaluated for all time points (on day 3, 15, 30).

### Statistics

Sample size calculation (*n* = 4) was assessed according to previous studies [22, 26, 34]. Primary outcomes were the characterization of the cellular reaction in terms of the induction of multinucleated giant cells (MNGCs) and vascularization. Secondary outcomes were signaling molecule expression (TRAP and CD-68, calcitonin receptor, MMP-9 and integrin-β3). All the output data were introduced into GraphPad Prism 7.0 software (GraphPad Software Inc., La Jolla, CA, USA) and statistically evaluated by one-way and two-way analysis of variance (ANOVA). The results were all expressed as the mean and standard deviation (SD) and depicted as column bars. Differences were considered statistically significant if the *p* values were *< 0.05 and highly significant if the *p* values were **< 0.01, ***< 0.001, and ****< 0.0001.

## Results

### Ex vivo analysis of the cellular interaction with Creos Xenoprotect (CXP)

**Fig. 1:**
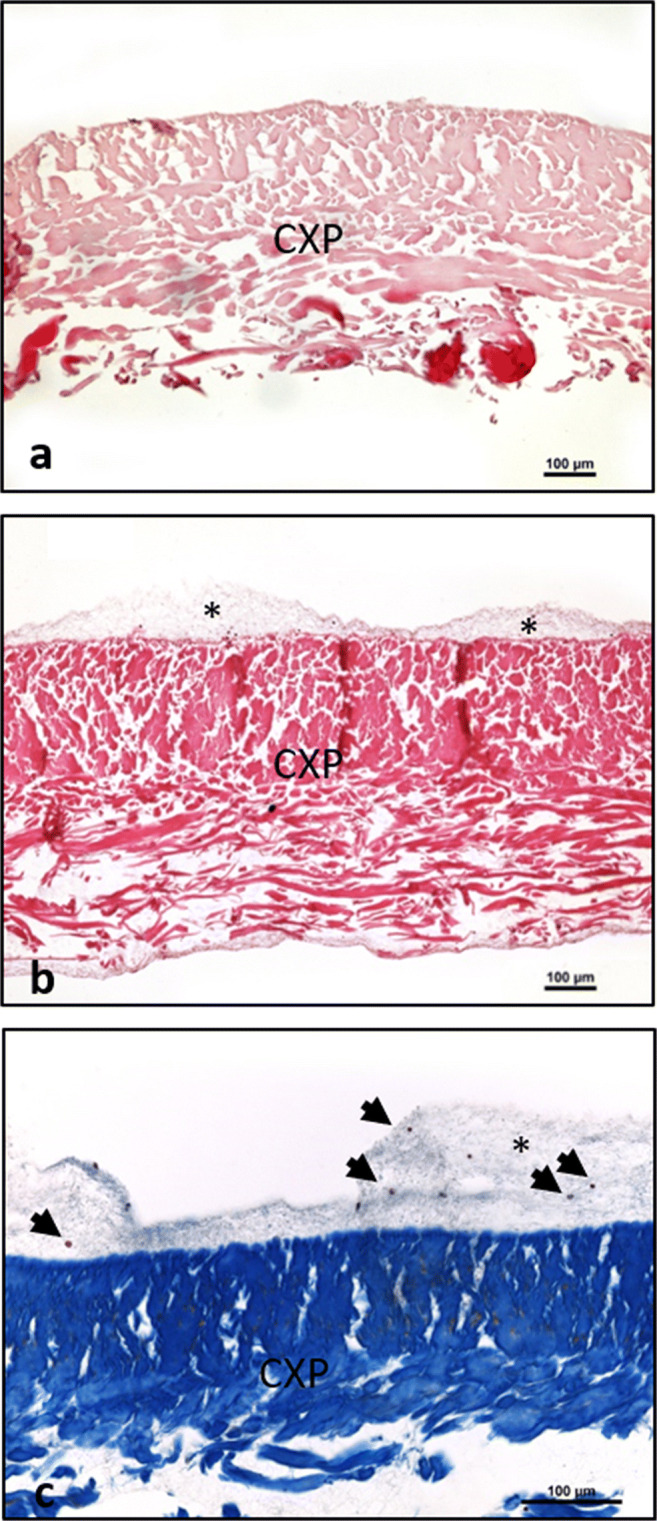
Ex vivo interaction between membrane Creos™ Xenoprotect (CXP), blood-derived cells, and fibrin from liquid-PRF. **a** A control cross section of the native membrane ex vivo, H and E staining; × 100 magnification. **b** CXP after liquid-PRF application. A fibrin clot is formed on the membrane surface (*); H and E staining; × 100 magnification. **c** High magnification of fibrin clot formation on the membrane surface (*) including blood cells (arrows). The membrane was not invaded by cells

Liquid-PRF, as a blood concentrate system containing a large number of platelets and leukocytes, was used to evaluate the cellular interaction and membrane permeability ex vivo over 15 min as previously described [26]. Fifteen minutes after application, the liquid-PRF formed a fibrin clot. Histological analysis showed that the membrane was not penetrated by the leukocytes and platelets from liquid-PRF (Fig. 1a–c). A fibrin clot including leukocytes and platelets was formed on both surfaces of the membrane (Fig 1b). However, membrane cross sections showed no cellular penetration into the membrane body in any of the evaluated CXP samples (Fig. 1c).

### Qualitative analysis of the in vivo cellular reaction

In the following section, the observed cellular reaction is described for both Creos™ Xenoprotect (CXP) and control group.

#### Control group

The sham-operated control group was used to mimic wound healing without biomaterial implantation as previously described [22, 23]. All animals survived the operation and the observation time period without any complications or atypical feeding or sleeping behaviors. No wound dehiscence, infection, or wound healing disorders were observed at any time point. Across the healing time points, i.e., day 3, 15, 30, only mononuclear cells (e.g., monocytes, macrophages, fibroblasts, lymphocytes) were observed within the wound healing region. No multinucleated giant cells (MNGCs) were detected at any time point.

#### Qualitative analysis of the in vivo cellular reaction to Creos™ Xenoprotect (CXP)

**Fig. 2:**
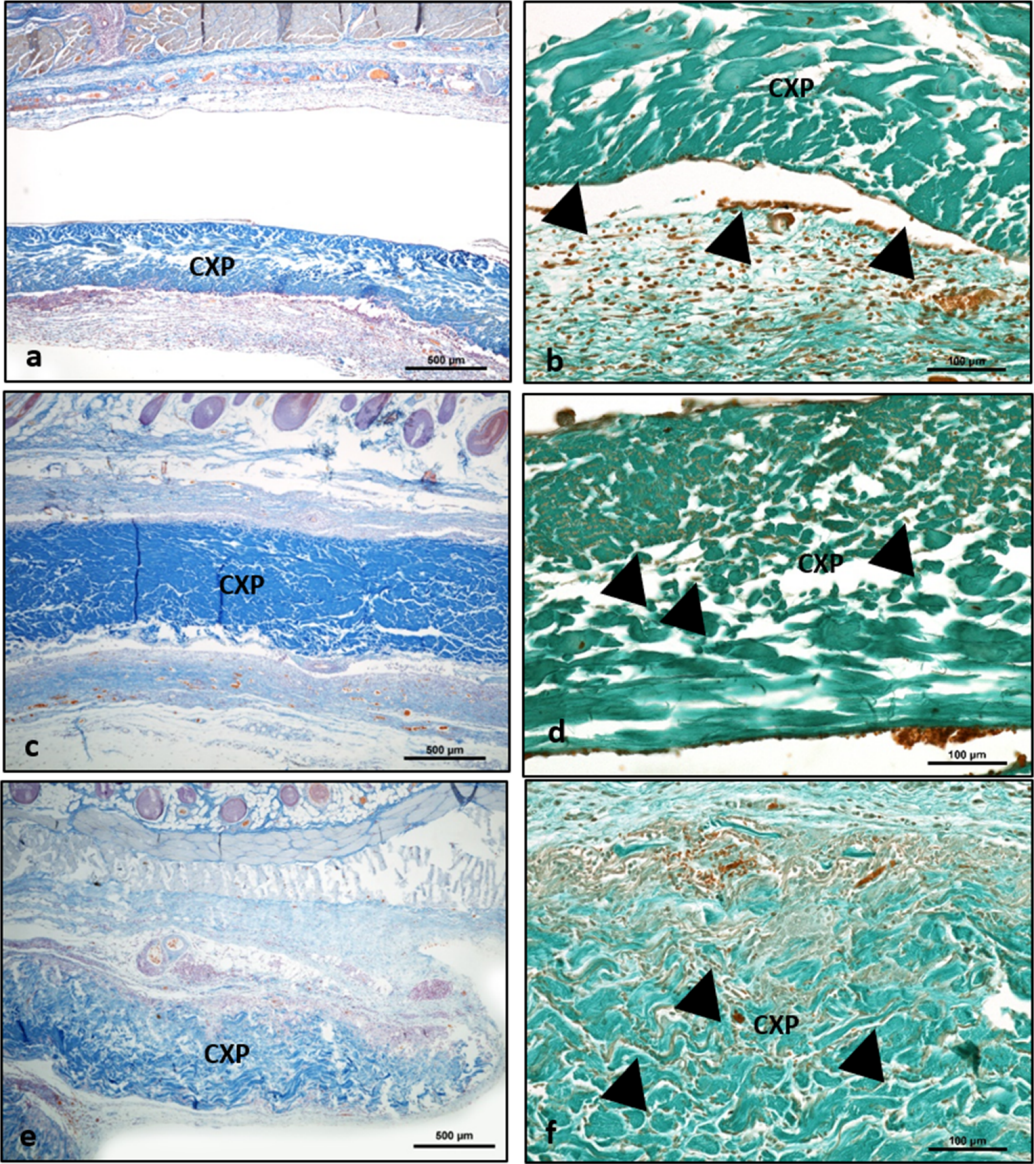
The cellular reaction to Creos™ Xenoprotect (CXP). **a** and **b** Day 3. **c** and **d** Day 15. **e** and **f** day 30. Left column azan staining; × 100 magnification. Right column Masson-Goldner staining; × 200 magnification. Arrows, peri-implantation tissue/cells

All animals survived the operation and the observation time period without any complications or atypical feeding or sleeping behaviors. No wound dehiscence, infection, or wound healing disorders were observed at any time point. The membrane was detectable within the implantation area after 3 days (Fig. 2a). A large number of mononuclear cells accumulated on the surface, including lymphocytes, monocytes, and macrophages. Some of the mononuclear cells expressed CD-68. Some cells started invading the CXP toward its center region. However, the central area was generally free of cells. New vessel formation was observed in the peri-implant region (Fig. 2b).

After 15 days, the number of mononuclear cells increased, illustrating a wall of inflammatory cells on the CXP surface (Fig. 2c). Thus, more cells invaded CXP toward its central region. At this time point, no capsule formation was observed. The membrane displayed a stable structure and maintained its integrity without allowing connective tissue ingrowth from the peri-implantation area (Fig. 2c). Single MNGCs could be detected on the biomaterial surface. Most of the induced MNGCs were TRAP negative. Additional vessels were detected in proximity to the membrane in comparison with the initial time point on day 3. However, no vessel formation was observed within the biomaterial body (Fig. 2d).

**Fig. 3:**
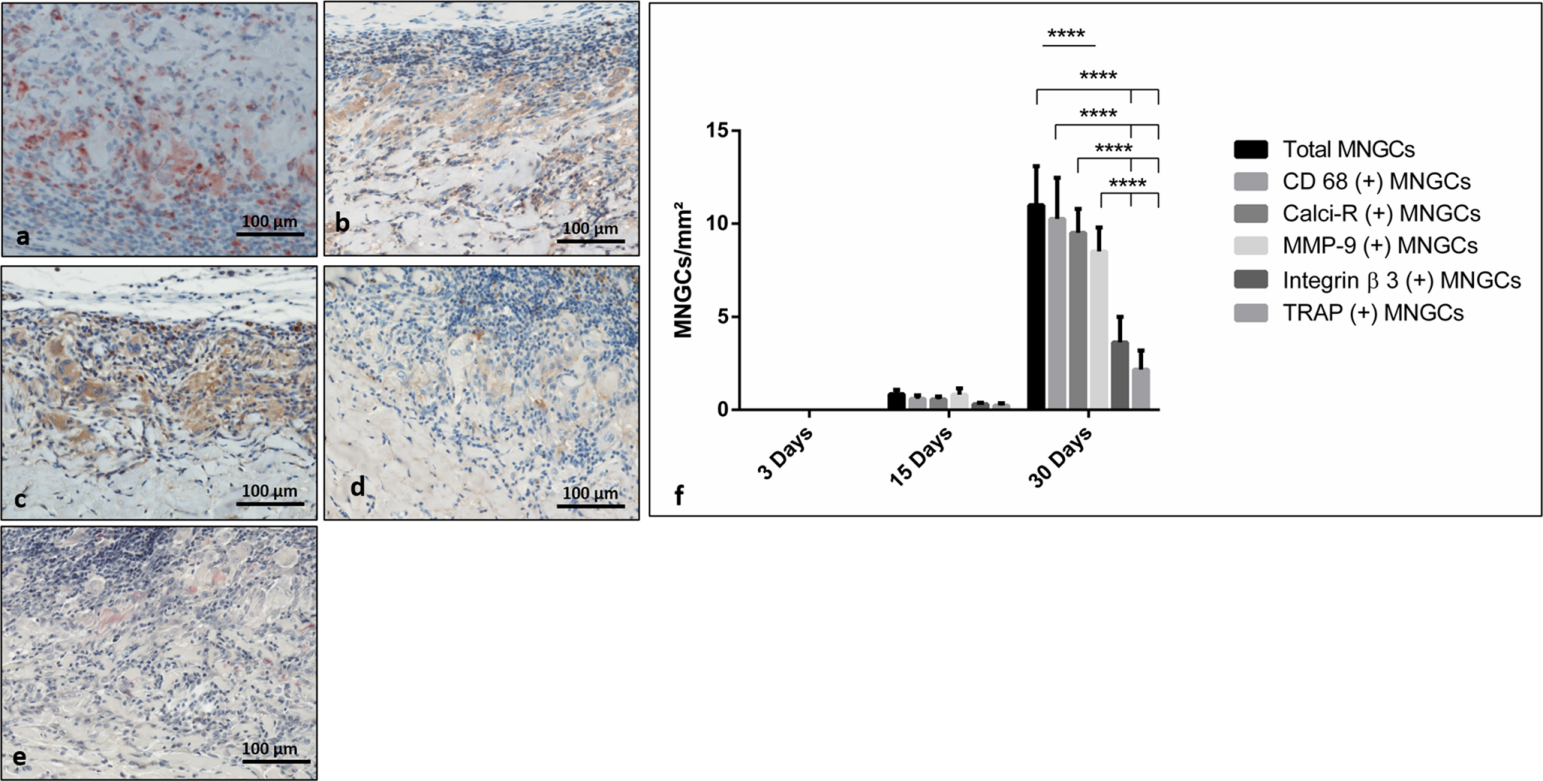
Immunhistological staining of the different expression markers in multinucleated giant cells (MNGCs). Positive cells are stained in red/brown. **a** Anti-CD-68. **b** Anti-calcitonin receptor. **c** Anti-MMP-9. **d** Anti-integrin-β3. **e** TRAP staining. **f** Statistical analysis of the histomorphometrically measured MNGC number per square millimeter. *p < 0.05; **p < 0.01; ***p < 0.001; ****p < 0.0001

On day 30 following implantation, the membrane was invaded by mononuclear cells that reached the central region. Immunohistological staining showed that some mononuclear cells within the implantation bed of CXP expressed CD-68. Compared with day 15, a greater number of MNGCs formed on the surface of CXP (Fig. 2e). These cells started to invade the membrane, followed by connective tissue ingrowth that progressed toward the central region of the membrane. The membrane showed a high rate of degradation and influx of connective tissue as markers of disintegration (Fig. 2f). Most of the induced MNGCs showed no TRAP and integrin-ß3 activity. In addition, vessel formation was observed within the superficial region of the membrane. However, a high expression of CD-68, calcitonin receptor, and MMP-9 was observed (Fig. 3a–e).

### Quantitative histomorphometric analysis

The comparative statistical analysis of the quantified data of material-induced multinucleated giant cells (MNGCs), number of cells expressing CD-68, and the vascularization rate is presented in the following section.

#### Material-induced multinucleated giant cells (MNGCs)

**Fig. 4:**
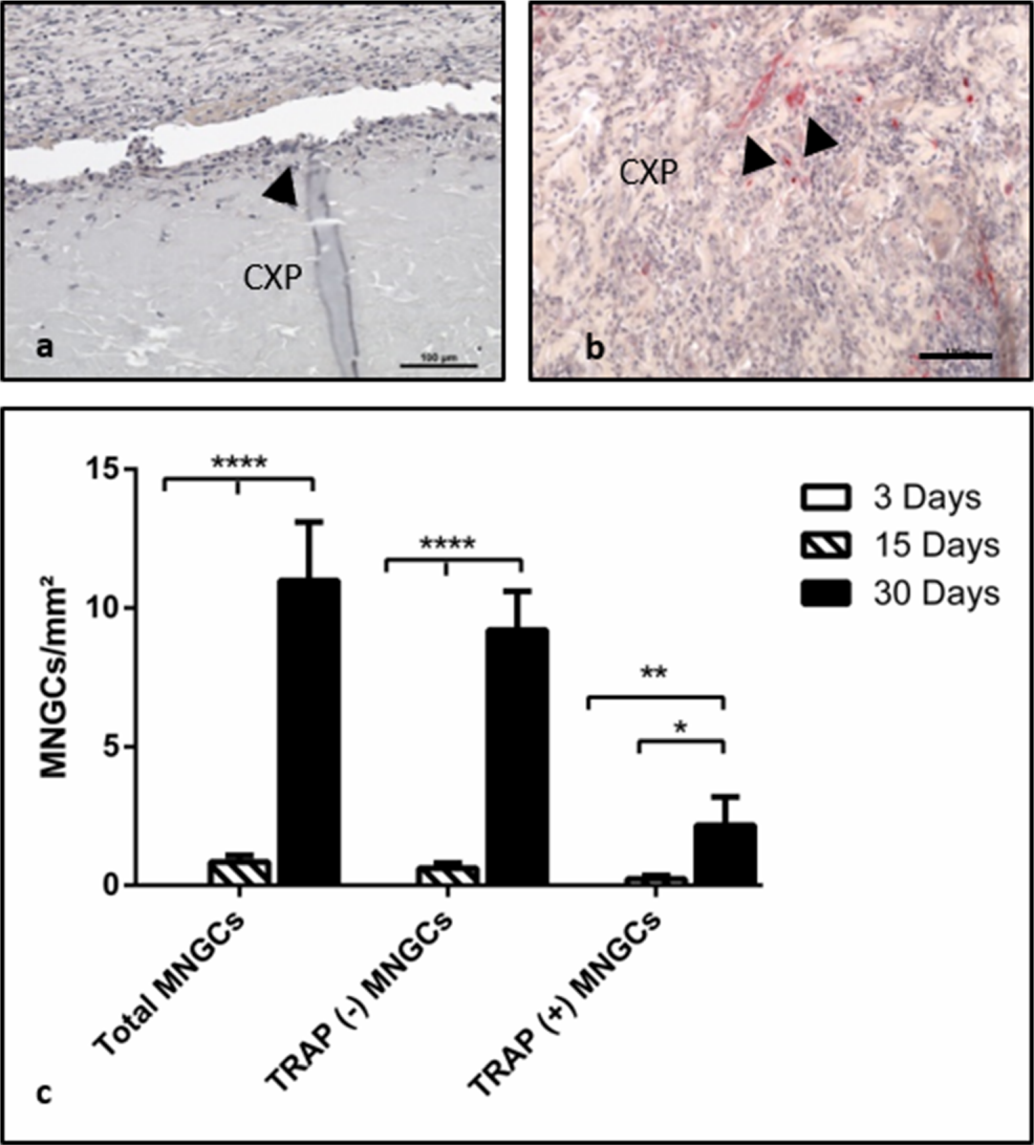
Biomaterial-induced multinucleated giant cells (MNGCs). **a**TRAP-negative MNGCs (arrowhead) on Creos™ Xenoprotect (CXP) surface on day 15. **b** TRAP-positive cells (arrowheads) invaded CXP on day 30. TRAP staining; × 200 magnification. **c** Statistical analysis of the histomorphometrically measured MNGC number per square millimeter. *p < 0.05; **p < 0.01; ***p < 0.001; ****p < 0.0001

In the control group, no MNGCs were observed at any time point. In the Creos™ Xenoprotect (CXP) test group, the membrane induced MNGCs starting on day 15 (Fig. 4a). Toward day 30 (Fig. 4b), the number of induced MNGCs per square millimeter increased significantly compared with the previous time points on day 15 (*p* < 0.0001) and 3 days (*p* < 0.0001) (Fig. 4c). On day 15, some MNGCs expressed TRAP. However, their number was not statistically significant compared with the TRAP-negative MNGCs. After 30 days, TRAP activity was detected in the analyzed material-induced MNGCs, but their number was significantly reduced (*p* < 0.0001) compared with the TRAP-negative MNGCs representative of most of the material-induced MNGCs at this time point (Fig. 4c).

MNGCs expressed different signaling molecules. Differences were detected especially on day 30. The number of MNGCs expressing CD-68 was the highest, followed by calcitonin receptor and MMP-9. However, the number of MNGCs expressing MMP-9 was significantly lower compared with the total number (**** *p* < 0.0001). Additionally, the number of MNGCs expressing TRAP and integrin-ß3 was the lowest. Both were significantly lower compared with the total MNGCs number (**** *p* < 0.0001), CD-68-positive MNGCs (**** *p* < 0.0001), calcitonin receptor (**** *p* < 0.0001), and MMP-9 (**** *p* < 0.0001) (Fig. 3f).

#### Vascularization pattern over time

The vessel density was evaluated histomorphometrically in the CXP group and control group over the evaluation time period of 30 days.

**Fig. 5:**
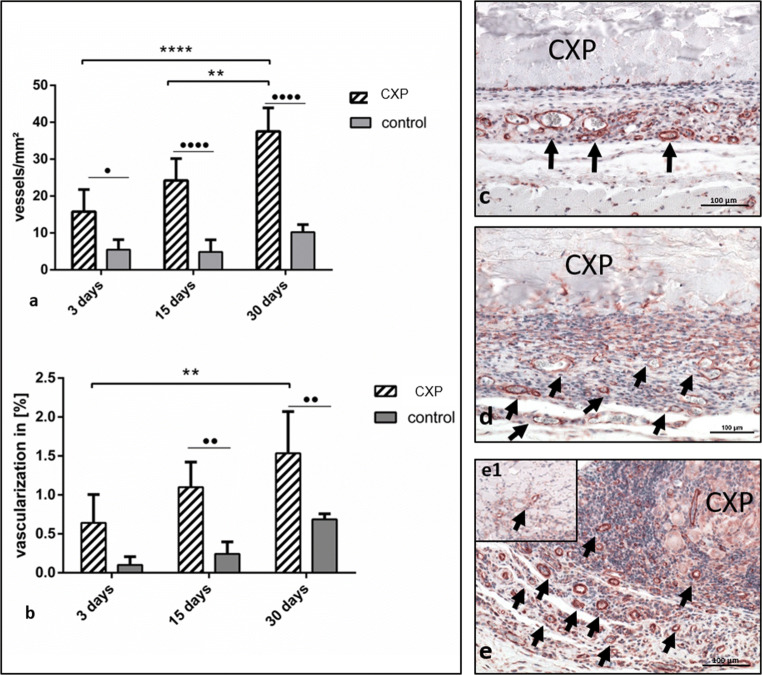
Vessel formation within Creos™ Xenoprotect (CXP). Statistical analysis of the histomorphometrically measured vessel number per square millimeter. *p < 0.05; **p < 0.01; ***p < 0.001; ****p < 0.0001. (**a**) Vessel density, (**b**) vascularization rate as a percent, (**c**–**e**) immunohistological staining of α-SMA marked vessels (arrows) (**c**) day 3, (**d**) day 15, and (**e**) and (**e1**) day 30. All images were captured at × 200 magnification

Intraindividual analysis revealed a progressive increase in vascularization in the CXP group in which the vessel density increased notably from day 3 to day 15. However, the difference was not statistically significant. Toward day 30, a significant increase in vessel density was observed compared with day 3 (*p* < 0.0001) and day 15 (*p* < 0.01) (Fig. 5a–e).

In the control group, the vessel density increased from day 3 to day 30 without a statistically significant difference.

On day 3, the interindividual analysis showed that the vessel density in the CXP group was significantly higher than that of the control group at the same time point (*p* < 0.05). Similarly, a significantly higher vessel density was detected in the CXP group on day 15 compared with the control group (*p* < 0.0001) on the same day. Finally, on day 30, the vessel density was higher in the CXP group than the control group, demonstrating a highly significant difference at that same time point (*p* < 0.0001) (Fig. 5a–e).

A similar pattern was observed when evaluating the percent vascularization. The percent vascularization increased progressively in both groups. The intraindividual analysis showed no statistically significant differences in the control group. However, the CXP group showed a significantly higher percentage of vascularization on day 30 compared with day 3 (*p* < 0.01) (Fig. 5b–e).

The interindividual analysis revealed significantly higher vascularization in the CXP group compared with the control group on both day 15 (*p* < 0.01) and day 30 (*p* < 0.01). However, no statistically significant difference was detected on day 3 (Fig. 5b–e).

#### CD-68 expression in mononuclear cells

**Fig. 6:**
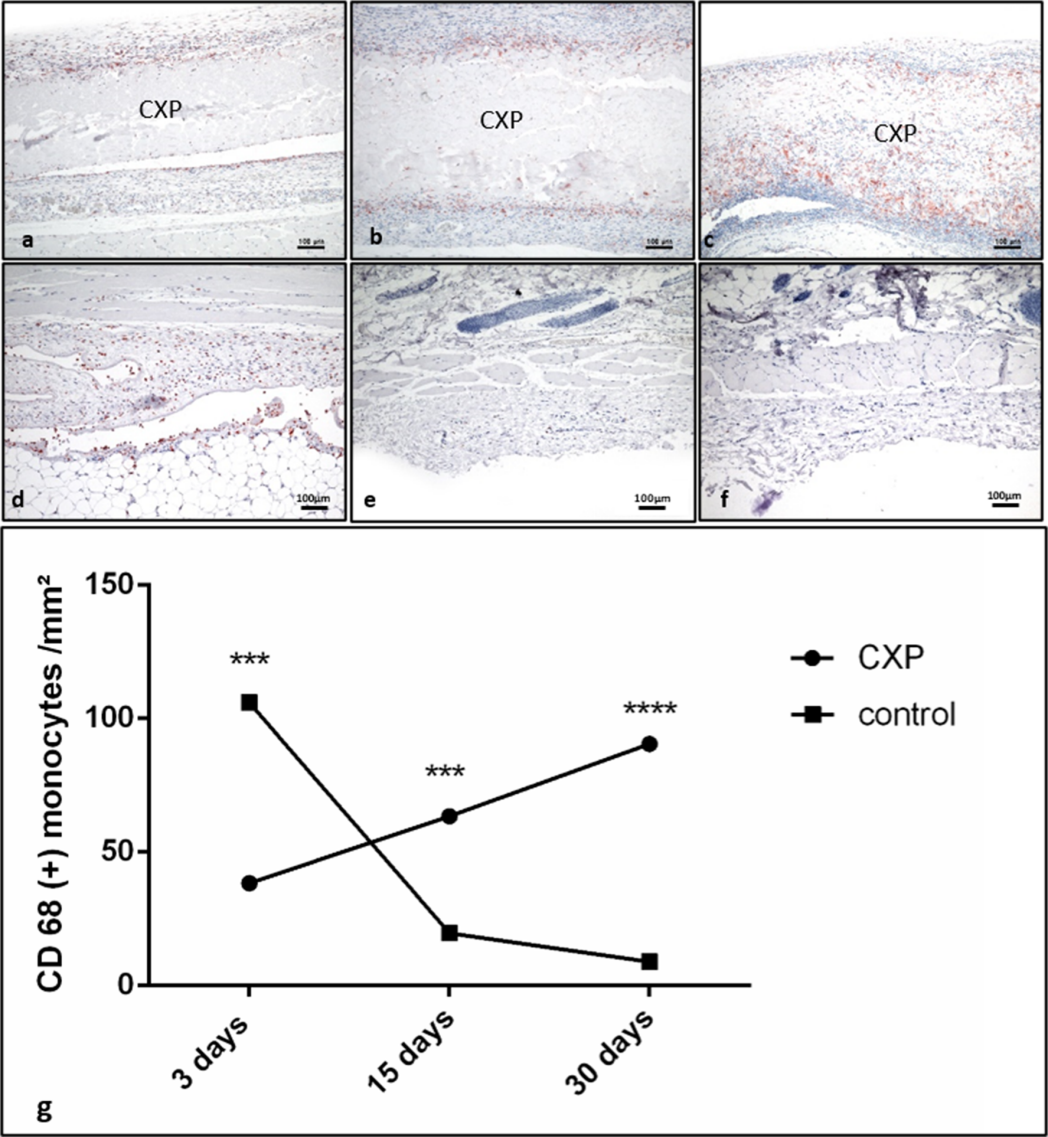
Immunohistological staining of CD-68-positive cells (**a**) day 3, (**b**) day15, and (**c**) day 30 in the Creos™ Xenoprotect (CXP) group and (**d**) day 3, (**e**) day 15, and (**f**) day 30 in the control group. All graphs were captured at × 100 magnification. **g** Statistical analysis of the histomorphometrically measured CD-68-positive mononuclear cell number per square millimeter. *p < 0.05; **p < 0.01; ***p < 0.001; ****p < 0.0001

Histomorphometric measurements of CD-68 expressing cells were calculated per square millimeter in the CXP (Fig. 6a–c) and control group (Fig. 6d–f).

On day 3, the number of CD-68-positive cells was significantly higher in the control group (*p* < 0.001) compared with the test group. Toward day 15, the number of CD-68-positive mononuclear cells was decreased in the control group. However, in the CXP test group, the number of CD-68-positive cells increased significantly. Consequently, a significantly higher cell number per square millimeter was detected in the CXP compared with the control group (*p* < 0.001). Thirty days after implantation, the number of CD-68-positive cells was further reduced compared with day 15 in the control group. By contrast, the CXP group showed a slight increase in CD-68-positive cells compared with day 15. The difference between the tested groups at this time point was statistically highly significant, demonstrating an increase in the CXP compared with the control group (*p* < 0.0001) (Fig. 6g).

## Discussion

In the last few years, the European market has experienced an influx of different collagen-based membranes and matrices that were introduced for applications in GTR and GBR [26]. Collagen-based biomaterials are derived from different origins, including allogeneic and xenogeneic materials. In addition, different harvesting species and compartments were used to manufacture well-engineered, biocompatible, and clinically practical membranes and matrices. The most common harvesting compartments are porcine pericardium, dermis, and skin [19]; bovine Achilles tendon [23]; and porcine tendon [22]. Collagen is a favorable material for application as a resorbable scaffold or membrane in tissue engineering [35] because it is a highly conserved protein that exists in most mammals and is therefore well tolerated as a naturally derived biomaterial [11]. Additionally, physiological native collagen exhibits good features to support cell migration, proliferation, and angiogenesis [36].

Although collagen-based biomaterials consist of the same construction unit, i.e., collagen, which is mostly preserved in its natural structure, different processing and purification methods are applied to eliminate pathogens and avoid the risk of xenotransplantation of immunologically active material [37]. Moreover, some manufacturers implement further techniques to reinforce the biomaterial stability by means of chemical or physical cross-linking methods [22]. These changes in the physical and chemical properties of materials have a prominent influence on the induced cellular reaction in vivo after biomaterial implantation into the host tissue [26]. Therefore, the present study aimed to analyze the cellular reaction to a novel non-chemically cross-linked collagen membrane to understand its pattern of regeneration and degradation. A wound healing model was used as a control to mimic physiological wound healing without the biomaterial. The authors believe that the most accurate control in this case is the physiological wound healing without biomaterial as demonstrated in previous studies [23]. It is not reasonable to compare different commercially available collagen-based biomaterials, as the manufacturing and processing methods significantly influence the cellular reaction as previously published [19, 38]. Therefore, to the best of our knowledge, no reliable parameters are presently known to characterize an appropriate control in terms of a collagen-based biomaterial. In this context, in this study, the physiological wound healing was considered as a control to assess biomaterial-related changes in relation to the physiological regeneration pattern. However, summarizing the reactions from different data can provide evidence to other researches. In this context, one of the limitations of the present study is the analysis of only one membrane in comparison with physiological healing. Additionally, the present data can be compared with previously published studies that used the same standardized model and evaluation methods but analyzed different collagen-based biomaterials.

This study presents two preclinical models to characterize the cell-matrix interaction ex vivo and in vivo. Ex vivo, a leukocyte and platelet-rich cell suspension (liquid-PRF) was used to mimic the first interaction between the Creos™ Xenoprotect (CXP) membrane and the host cells, i.e., blood cells, as previously described [22, 23].

The ex vivo results showed that the blood-derived leukocytes and platelets accumulated on the membrane surface and could not penetrate the biomaterial. Moreover, a fibrin clot was formed on the biomaterial surface, including the blood-derived cells. Similar results were observed in previous studies that used liquid-PRF to investigate other collagen-based membranes of bovine and porcine origin. Regardless of the rate of porosity, the membranes were occlusive to blood cells in liquid-PRF [22, 23]. PRF was used as a cell suspension to evaluate the initial biomaterial interaction with blood cells and the biomaterial capacity of absorbing blood components. In this case, only one biomaterial was tested to compare the in vivo and the ex vivo results in order to understand the absorption capacity of this specific biomaterial.

The ex vivo results were in accordance with the in vivo observations 3 days after implantation. In this case, the membrane induced only physiological mononuclear cells (e.g., monocytes, macrophages, lymphocytes), which were also observed in the control group and did not allow the cells to enter the biomaterial body. However, toward day 30 following implantation, the membrane induced a large number of multinucleated giant cells (MNGCs), which were first only located on the biomaterial surface (day 15) but then proceeded to infiltrate the biomaterial body (day 30) along with the mononuclear cells and peri-implantation connective tissue. Remarkably, the control group did not show any induction of MNGCs at any time point. This observation supports the suggestion that MNGCs are rather biomaterial-related cells that are not involved in physiological wound healing and regenerative processes [26]. Biomaterial-induced MNGCs are frequently observed in the implantation bed of different biomaterials [26, 39]. In addition, common surface molecules and secretors are found between the biomaterial-induced MNGCs and other pathological MNGC types that are found, for example, in sarcoidosis [26].

Previous in vitro and in vivo studies have shown that MNGCs are formed by the fusion of macrophages after their frustrated attempt to degrade the biomaterial in a process called “frustrated phagocytosis” as an indication of a foreign body reaction [22, 23, 40]. However, MNGC induction and formation depend primarily on the biomaterial surface characteristics and, thereby, the adhesive molecules expressed by the cells [41]. Interestingly, the present study demonstrated a dynamic decrease in the number of macrophages (CD-68-positive mononuclear cells) in the control group as a physiological life cycle of macrophages [42]. In contrast, the CXP group showed a significant increase over of persisting macrophages in the implantation bed. This phenomenon is associated with a chronic inflammation as one sign of a foreign body reaction [43]. Consequently, persisted macrophages fused to form biomaterial-induced MNGCs as observed in the present results starting with day 15 until day 30. The present findings are in agreement with a previous in vivo study investigating a similar membrane [23]. Thus, both studies observed the formation of MNGCs in response to collagen membrane CXP. The MNGCs observed herein were further characterized as TRAP-positive and TRAP-negative MNGCs. TRAP-positive MNGCs were detected on day 30, together with obvious biomaterial disintegration, which indicated a loss of integrity, fragmentation, and a loss of the initial structure. Thus, peri-implantation connective tissue was able to find its way into the central region of the membrane between membrane fragments. These observations are supported by a recent in vitro study, which have shown that both types of TRAP expression are associated with collagen degradation [44]. These findings showed that the biomaterial-induced TRAP-positive MNGCs in this case were associated with a high inflammatory reaction. TRAP is a degrading enzyme that exists in 2 isoforms, type 5a and type 5b, that have been suggested to have different functions [44–46]. It was first described in osteoclasts [47, 48]; however, recent studies have shown that TRAP may be considered as a pro-inflammatory signaling molecule [49, 50]. Similar findings were observed when analyzing the expression of MNGCs in inflammatory diseases such as sarcoidosis. TRAP type 5a was highly expressed in MNGCs and macrophages of sarcoidosis tissue. This study also suggested that TRAP type 5a is derived from systemic inflammatory of macrophages and thereby may be a biomarker of inflammation.

Another interesting finding of the present study was the correlation between the number of MNGCs and the rate of vascularization. With an increased number of MNGCs, a high vascularization rate was measured on day 15 and 30, which was significantly higher than in the control group, i.e., not comparable to the physiological vascularization that occurs during wound healing (supraphysiological). The large number of vessels was potentially induced by the pro-inflammatory microenvironment resulting from the persistence of CD-68-positive cells and induction of MNGCs. In a previous in vivo study, biomaterial-induced MNGCs were shown to produce vascular endothelial growth factor (VEGF) in vivo [31]. Additionally, the number and kinetics of the biomaterial-induced MNGCs have been shown to directly influence its degradation pattern. A recent study analyzed a bovine-derived collagen-based membrane gained from Achilles tendon in the same implantation model. The bovine-derived membrane also induced MNGCs but in a different pattern. In this case, there was no statistically significant increase of the MNGCs number from day 15 to 30. Thereby, the membrane did not undergo a total disintegration, as it was observed by the present membrane [23]. These results show that even though most of the clinically used membranes are collagen-based, different in vivo reactions are observed according to the biomaterial-specific physicochemical properties, manufacturing technique, surface morphology and structure. In this context, our group performed a standardized series of studies to classify different polymeric biomaterials according to the in vivo induced cellular reaction. The results showed that there are three different cellular reaction patterns based on the biomaterial properties [51]. Class I incudes biomaterials that do not induce MNGCs at any time point; class II includes biomaterials that induce MNGCs with a constant number over 30 days; and class III includes biomaterials that induce MNGCs with continuous increasing number over 30 days [51]. Based on this classification, the here analyzed membrane fits in class III.

Previously, biomaterial-induced MNGCs were considered as osteoclasts, because of their morphological similarities. However, biomaterial-induced MNGCs and osteoclasts express some signaling molecules in common such as CD-68 [21]. A previous histological study that evaluated the expression pattern of biomaterial-induced MNGCs in human biopsies showed that biomaterial-induced MNGCs show a high expression of inflammatory signaling molecules such as Cox-2 or CCR-7 and rather low expression of other markers such as CD-163 or CD-206 [52]. Additionally, osteoclasts are known to express markers like RANKL, calcitonin receptor, integrin-ß3, and TRAP. However, little is known about the expression of these markers in the biomaterial-induced MNGCs. A recent in vitro study showed that calcitonin receptors show rather a low expression in biomaterial-induced MNGCs [21]. By contrast, the present study showed a high expression of calcitonin receptor in the biomaterial-induced MNGCs. Different studies showed that the high expression of calcitonin receptor inhibits the activity of osteoclasts [53]. It may be presumed that a similar effect of this molecule will play a role in the biomaterial degradation. However, further research is needed to understand this mechanism. Additionally, biomaterial-induced MNGCs highly expressed MMP-9, a matrix metalloprotease. MMP-9 was found to be expressed in both osteoclasts [54], foreign body MNGCs, and disease-related MNGCs such as tuberculosis [55, 56]. In addition, the upregulation of MMP-9 in an inflammatory process has been shown to play an important role in the fusion of macrophages to form MNGCs [55]. This molecule has been additionally shown to be involved in collagen degradation in different localization [57, 58]. In this context, the high expression of MMP-9 in the biomaterial-induced MNGCs is a potential marker for their role in the biomaterial degradation.

A further study characterized the type of MNGCs according to the expressed type of integrin. Integrins ß1 and ß2 have been described in the process of foreign body MNGC fusion, while integrin-ß3 is rather expressed in osteoclasts [59]. The present finding confirms these data, as the biomaterial-induced MNGCs in this study showed a rather low expression of integrin-ß3. Similar findings were observed in the case of TRAP expression. Based on these data, the present study showed that biomaterial-induced MNGCs may have similarities with osteoclasts, especially in the inflammatory markers (CD-68, MMP-9), but not in marker that are related to bone adhesion and resorption (integrin-ß3, TRAP).

To date, little is known about the role of biomaterial-induced MNGCs in the process of regeneration [25]. One function may be to support biomaterial degradation in resorbable biomaterials. However, other collagen-based membranes, such as non-cross-linked collagen I and III porcine-derived membrane and matrix, were evaluated in vivo using similar subcutaneous implantation models as demonstrated in the present study. These collagen-based materials did not induce any MNGCs at any time point over an observation time of 60 days [20, 34]. They induced only mononuclear cells, which are physiologically involved in the process of regeneration, such as macrophages, lymphocytes, and fibroblasts; preserved their native structure over the study period; and were well integrated into the host tissue after 60 days. Additionally, their slow degradation without a loss of function was primarily associated with macrophages. These observations show that biomaterials do not induce foreign body MNGCs per se and that it is possible to produce biomaterials that induce only mononuclear cells as a physiological reaction. At this point, the question arises whether MNGCs have any contribution to the process of regeneration and whether clinicians should accept this reaction in clinical applications.

The disintegration of the membrane evaluated herein after 30 days showed that the membrane might be alternately used for indications for which rapid biomaterial degradation and induction of a high rate of vascularization are required rather than for indications in which long-term stability is necessary.

The present study also demonstrated that the wound healing microenvironment after biomaterial implantation is more complex than physiological wound healing. The present study analyzed only one membrane in comparison with the physiological wound healing. Because of the high number of different parameters that may influence the cellular reaction, it would be interesting to manufacture the same collagen membrane and modify only one parameter such as cross-linking to be able to separately evaluate this parameter in a more standardized and systematic study. Collagen-based membranes were previously considered to serve as an inactive barrier to separate two types of tissue in terms of GTR or GBR. However, recent in vivo studies have shown that the applied collagen membrane significantly influences the underlying augmentation area [16]. A previous in vivo study investigated the same collagen membrane that was evaluated in this study (CXP) using a bone defect model, further comparing it with a porcine-derived collagen membrane [60]. The results revealed different rates of newly formed bone according to the implanted collagen membrane, although both membranes were of porcine origin [60]. These outcomes demonstrate that despite of the identical origin, different collagen membranes contribute differently to the GBR process based on their interaction with the surrounding tissue and the induced cellular reaction. Thus, understanding the cellular reaction to biomaterials is essential to assess their suitability for specific clinical indications. However, further controlled clinical studies are needed to outline the potential benefit to the specific group of biomaterials in the respective clinical indications.

Taken together, the present study showed that biomaterial implantation leads to changes in the physiological cellular kinetics of wound healing in a biomaterial-specific cellular reaction. Ex vivo and in vivo interactions with the host tissue showed that the CXP biomaterial investigated herein was initially occlusive to host tissue cells, but the induction and accumulation of MNGCs led to its disintegration after 30 days. The present findings mandate further characterization of the biomaterial-induced MNGCs to outline their potential functions in the process of regeneration.

## Conclusion

This study characterized the cellular reaction to a novel collagen-based membrane of porcine origin ex vivo and in vivo in comparison with physiological wound healing. The tissue response revealed an initial accumulation of mononuclear cells, such as monocytes, macrophages, lymphocytes, and fibroblasts, at the early time point in both ex vivo and in vivo examinations. From day 15 onward, the membrane induced MNGCs that were first localized on the membranes surface and then proceeded toward the central membrane region, followed by connective tissue influx from the peri-implantation region. During physiological wound healing, no MNGCs were observed at any time point. After material implantation, a dynamic change was observed in the number of CD-68-positive cells (macrophages), which correlated with the induction of MNGCs. MNGCs highly expressed CD-68, calcitonin receptor, and MMP-9 but not integrin-β3 and TRAP. These findings support that biomaterial-induced MNGCs are rather a sign of a foreign body reaction. The induced MNGCs led to membrane disintegration after 30 days and a loss of integrity. Thus, the present study calls for further preclinical and controlled clinical research to characterize the biomaterial-induced MNGCs and to elucidate their potential function in the degradation process.
